# Double-Blind Placebo-Controlled Treatment Trial of *Chlamydia trachomatis* Endocervical Infections in Pregnant Women

**DOI:** 10.1155/S1064744997000057

**Published:** 1997

**Authors:** David H. Martin, David A. Eschenbach, Mary Frances Cotch, Robert P. Nugent, A. Vijaya Rao, Mark A. Klebanoff, Yu Lou, Philip J. Rettig, Ronald S. Gibbs, Joseph G. Pastorek II, Joan A. Regan, Richard A. Kaslow

**Affiliations:** 1 Department of Medicine and Obstetrics and Gynecology Louisiana State University New Orleans LA USA; 2 Department of Obstetrics and Gynecology University of Washington Seattle WA USA; 3 National Institute of Allergy and Infectious Diseases Bethesda MD USA; 4 National Institute of Child Health and Human Development Bethesda MD USA; 5 Research Triangle Institute Research Triangle Park NC USA; 6 Departments of Pediatrics and Obstetrics and Gnecology University of Oklahoma Oklahoma City OK USA; 7 Department of Obstetrics and Gnecology University of Texas San Antonio TX USA; 8 Department of Pediatrics Columbia University New York NY USA; 9 Department of Obstetrics and Gynecology University of Colorado Denver CO 80262 USA; 10 NIH, NICHD, PAMA Branch 6100 Executive Boulevard Room 4BllC Bethesda MD 20892 USA

## Abstract

*Objective:* The purpose of this study was to determine if treatment of pregnant women with *Chlamydia trachomatis* infection would lower the incidence of preterm delivery and/or low birth weight.

*Methods:* Pregnant women between the 23rd and 29th weeks of gestation were randomized in double-blind fashion to receive either erythromycin 333 mg three times daily or an identical placebo. The trial continued until the end of the 35th week of gestation.

*Results:* When the results were examined without regard to study site, erythromycin had little impact on reducing low birth weight (8% vs. 11%, *P* = 0.4) or preterm delivery (13% vs. 15%, *P* = 0.7). At the sites with high persistence of *C. trachomatis* in the placebo-treated women, low birth weight infants occurred in 9 (8%) of 114 erythromycin-treated and 18 (17%) of 105 placebo-treated women (*P* = 0.04) and delivery <37 weeks occurred in 15 (13%) of 115 erythromycin-treated and 18 (17%) of 105 placebo-treated women (*P* = 0.4).

*Conclusions:* The results of this trial suggest that the risk of low birth weight can be decreased by giving erythromycin to some women with *C. trachomatis*. Due to the high clearance rate of *C. trachomatis* in the placebo group, these data do not provide unequivocal evidence that erythromycin use in all *C. trachomatis*-infected women prevents low birth weight.


Infectious Diseases in Obstetrics and Gynecology 5:10–17 (1997)
© 1997 Wiley-Liss, Inc.

### Double-Blind Placebo-Controlled Treatment Trial of *Chlamydia trachomatis* Endocervical Infections in Pregnant Women

David H. Martin,[1] David A. Eschenbach,[2] Mary Frances Cotch,[3] Robert P. Nugent,[4]* A. Vijaya Rao,[5] Mark A. Klebanoff,[4] Yu Lou,[5] Philip J. Rettig,[6] Ronald S. Gibbs,[7] Joseph G. Pastorek II,[1] Joan A. Regan,[8] and Richard A. Kaslow[3]

[1]*Department of Medicine and Obstetrics and Gynecology, Louisiana State University, New Orleans, LA*
[2]*Department of Obstetrics and Gynecology, University of Washington, Seattle, WA*
[3]*National Institute of Allergy and Infectious Diseases, Bethesda, MD*
[4]*National Institute of Child Health and Human Development, Bethesda, MD*
[5]*Research Triangle Institute, Research Triangle Park, NC*
[6]*Departments of Pediatrics and Obstetrics and Gynecology, University of Oklahoma, Oklahoma City, OK*
[7]*Department of Obstetrics and Gynecology, University of Texas, San Antonio, TX*
[8]*Department of Pediatrics, Columbia University, New York, NY*

### Abstract

*Objective:* The purpose of this study was to determine if treatment of pregnant women with *Chlamydia trachomatis* infection would lower the incidence of preterm delivery and/or low birth weight.

*Methods:* Pregnant women between the 23rd and 29th weeks of gestation were randomized in double-blind fashion to receive either erythromycin 333 mg three times daily or an identical placebo. The trial continued until the end of the 35th week of gestation.

*Results:* When the results were examined without regard to study site, erythromycin had little impact on reducing low birth weight (8% vs. 11%, $P = 0.4$) or preterm delivery (13% vs. 15%, $P = 0.7$). At the sites with high persistence of *C. trachomatis* in the placebo-treated women, low birth weight infants occurred in 9 (8%) of 114 erythromycin-treated and 18 (17%) of 105 placebo-treated women ($P = 0.04$) and delivery <37 weeks occurred in 15 (13%) of 115 erythromycin-treated and 18 (17%) of 105 placebo-treated women ($P = 0.4$).

*Conclusions:* The results of this trial suggest that the risk of low birth weight can be decreased by giving erythromycin to some women with *C. trachomatis*. Due to the high clearance rate of *C.*

Contract grant sponsor: National Institute of Child Health and Human Development; Contract grant numbers: HD-3-2832 through HD-3-2836. Contract grant sponsor: National Institute of Allergy and Infectious Diseases; Contract grant number: AI-4-2532.

The authors are members of the Vaginal Infections and Prematurity (VIP) Study Group. Other members of the VIP Study Group are: Sumner J. Yaffe, Charlotte S. Catz, George G. Rhoads (now at the University of Medicine and Dentistry of New Jersey), Donald McNellis, Heinz W. Berendes, National Institute of Child Health and Human Development; Robert Edelman (now at the University of Maryland), William C. Blackwelder, George F. Reed, National Institutes of Allergy and Infectious Diseases; W. Kenneth Poole, Research Triangle Institute; Luann Wenthold, Department of Obstetrics and Gynecology, Louisiana State University; Molly Fischer, Department of Obstetrics and Gynecology, University of Washington; Arlene Meier, Departments of Pediatrics and Obstetrics and Gynecology, University of Oklahoma; Kathleen Lipscomb, Department of Obstetrics and Gynecology, University of Texas, San Antonio; Kim Geromanos, Department of Pediatrics, Columbia University.

Dr. Ronald S. Gibbs is now at the Department of Obstetrics and Gynecology, University of Colorado, Denver, CO 80262.

*Correspondence to: Dr. Robert P. Nugent, NIH, NICHD, PAMA Branch, 6100 Executive Boulevard, Room 4B11C, Bethesda, MD 20892.

Received 19 January 1997

Accepted 31 March 1997

***trachomatis* in the placebo group, these data do not provide unequivocal evidence that erythromycin use in all *C. trachomatis*-infected women prevents low birth weight. Infect. Dis. Obstet. Gynecol. 5:10–17, 1997.** © 1997 Wiley-Liss, Inc.

KEY WORDS
chlamydial infection; premature delivery; low birth weight

The prevalence of *Chlamydia trachomatis* varies widely depending upon the population, with 5–15% of pregnant women being infected.[1–3] During pregnancy, *C. trachomatis* is isolated 4–8 times more frequently than *Neisseria gonorrhoeae*.[2,4,5] *C. trachomatis* is particularly common among adolescents and individuals from low socioeconomic groups,[6,7] and it has been associated with adverse pregnancy outcomes in most, but not all, reports.[1,2,4,6,8,9]

Treatment of *C. trachomatis* in pregnancy has been associated with improved pregnancy outcome. In one study, the rate of abnormal pregnancy outcome was significantly lower in women with *C. trachomatis* who were treated with erythromycin over a 20 month study period compared to the rate in untreated women examined during the 16 month interval preceding the study period.[10] Furthermore, women infected with *C. trachomatis* who were treated had the same frequency of abnormal outcome as did uninfected women.[10] In another study, fewer abnormal pregnancy outcomes occurred among *C. trachomatis*-positive women who were effectively treated than among those who remained culture positive.[11] Neither of these studies used a randomized or blinded treatment protocol nor did they account for other bacteria or obstetrical factors associated with poor pregnancy outcome.

We report the results of a randomized placebo-controlled double-blinded trial of erythromycin treatment of *C. trachomatis* infection during pregnancy. Our study was able to adjust for the influence of demographic, sexual, and obstetrical factors as well as the presence of other potentially pathogenic organisms isolated from the lower genital tract.

### SUBJECTS AND METHODS

### Study Design

As part of the Vaginal Infection and Prematurity (VIP) Study, pregnant women were enrolled from seven institutions utilizing six antepartum clinics (Harlem Hospital, New York, NY; Columbia University, New York, NY; Louisiana State University and Tulane University, New Orleans; University of Oklahoma, Oklahoma City; University of Texas Health Science Center, San Antonio; and University of Washington, Seattle). A uniform protocol had been agreed upon, including a standardized questionnaire and common laboratory methods. Women seeking prenatal care between the 23rd and 26th completed weeks of pregnancy were screened for eligibility to enter the observational phase of the study. Women were eligible if they were ≥16 years old, were free of medical complications related to premature delivery, and were not taking selected medications. Details of inclusion and exclusion criteria have been published previously.[12,13] Research personnel obtained demographic, obstetric, sexual, contraceptive, and drug use history on standardized coded forms; performed a standardized physical and pelvic examination; and collected urogenital cultures in a standard manner. At delivery the medical records of the mother and infants were reviewed and information on intervening therapy, including non-trial antibiotic use, was abstracted.

Women identified as colonized with *Ureaplasma urealyticum*, group B streptococci, and/or *C. trachomatis* were considered for randomization into the clinical trial. The clinical trial results for women positive for *U. urealyticum* but negative for group B streptococci and *C. trachomatis* have been published,[13] as have the results for women with group B streptococci.[12]

### Collection of Urogenital Specimens

Following the insertion of sterile cotton swabs into the endocervix to obtain specimens for Gram's stain, and for *N. gonorrhoeae* and group B streptococcal culture, a sterile Dacron swab with a plastic shaft was then inserted within the endocervical canal and rotated for several seconds to obtain a specimen for *C. trachomatis* culture. The swab tip was broken off in a vial containing 1.5 ml of phosphate buffered 0.2 M sucrose solution. The specimen was maintained at refrigerator temperature until it reached the laboratory later that day. Details of specimen collection methods for the other microorganisms have been published.[13]

### Culture Methods

The protocol for *C. trachomatis* culture followed by all centers was as follows: the swab was removed from the transport vial and a sample of the carrier media was inoculated onto McCoy cell monolayers grown in one dram shell vials at all centers as described[14] except for the University of Washington, which used 96-well microtiter plates.[15] At least two monolayers were inoculated for each specimen. Vials or plates were then centrifuged for 1 h at 1,000–2,500 *g*. After replacement of the inoculum with cell culture media, vials and/or plates were incubated at 35°C for 48–68 h. One coverslip or one well was then stained with fluorescein isothiocyanate-conjugated *C. trachomatis*-specific monoclonal antibody and read with a fluorescent microscope. For positive specimens, *C. trachomatis* inclusions in 10–20 random microscopic fields were counted to semiquantitate the number of viable organisms in each specimen. For negative specimens, the cells were scraped off the bottom of the second vial or well using a pipette tip and inoculated onto fresh monolayers. The culture procedure was then repeated. A specimen was considered negative only if inclusions were not observed in monolayers inoculated with the passed specimen.

### Clinical Trial Methods

Women from whom *C. trachomatis* was recovered in the observational study were eligible to participate in the clinical trial. However, women receiving antibiotics since the screening examination, who were allergic to erythromycin or receiving theophylline were ineligible. Women with positive screening cultures for *N. gonorrhoeae* or $>10^5$ microorganisms/ml of urine were treated and thus ineligible for the trial.

Eligible women who agreed to participate in the clinical trial entered a 1 week placebo run-in.[16] Those who took less than two-thirds of the allotted placebo pills during the run-in, who did not return to the clinic or refused further participation were not randomized. Patients who successfully completed the run-in, had none of the exclusion criteria, and were <30 weeks gestation were randomized as described previously.[13] The randomization scheme stratified women by study site and microorganism combination to allow for subgroup-specific analyses. Participants were treated with either erythromycin base (333 mg) or identical-appearing placebo tablets 3 times daily from blister packs containing 21 pills each. The erythromycin and placebo were supplied by the Upjohn Company (Kalamazoo, MI).

As a result of concerns that a daily dose of 2 g of erythromycin would be poorly tolerated over 6 weeks, the lower 1 g dose was chosen. Earlier reports suggested that a 1 g daily dose for 6 weeks in the third trimester decreased the rate of low birth weight infants among women colonized with genital mycoplasmas.[17] Previous experience of the investigators suggested that 500 mg erythromycin taken twice daily for 14 days effectively treated endocervical *C. trachomatis* in pregnant women (Martin and Eschenbach, unpublished data). Treatment of partners was recommended, but therapy was given to the study participants until the completion of the 35th week of pregnancy to reduce the likelihood of reinfection.

Women were evaluated at each regular antenatal visit, at which time compliance (by pill count) and side effects were recorded by a research nurse using a standardized questionnaire, used blister packs were collected, and additional medication was supplied.

### Quality Control/Drug Efficacy Issues

Repeat cultures were obtained 2–4 weeks after enrollment from the first 100 women enrolled into the clinical trial at each study site. In addition, a random sample of 12% of all study participants was selected to have repeat cultures (6% at 31–33 weeks and 6% at 34–36 weeks gestation). Repeat cultures also were obtained from women admitted for pregnancy complications at <37 weeks gestation (premature labor, rupture of membranes) and from all women admitted to the hospital in term labor during weekday daytime hours.

Two methods were used for quality control of *C. trachomatis* cultures. Each study site in turn sent 5 "unknowns" that included both positive and negative specimens to the other centers. In addition, a random sample of women had duplicate *C. trachomatis* specimens obtained and submitted to the laboratories for culture. Different study numbers were assigned to blind laboratory personnel to the duplicate specimens.

### Definitions

Gestational age at study entry was estimated from the last menstrual period, results of the first pelvic examination, onset of fetal heart tone, and ultrasound data when available. Gestational age at delivery was calculated from the entry estimate and the time to delivery. All women had pertinent pregnancy, labor, and delivery information collected including the use of non-trial antibiotics. Premature rupture of membranes was defined as membrane rupture before the onset of regular uterine contractions.

Antibiotics considered effective against *C. trachomatis* included erythromycin, ampicillin, tetracycline, oral sulfa preparations, and penicillin VK. Antibiotics considered ineffective included metronidazole, cephalosporins, nitrofurantoin, penicillins other than VK or ampicillin, and vaginal sulfa cream.

### Post-Delivery

Trial participants with *C. trachomatis* were retreated with doxycycline, tetracycline, or erythromycin immediately postpartum, regardless of which trial medication they received. Infants were either treated empirically after delivery or were followed, cultured at their first postnatal visit, and treated with antibiotics if indicated.

### Statistical Analysis

Categorical variables were compared using the chi-square test or Fisher's exact test.[18] Significance was defined as a two-tailed $P < 0.05$. All calculations were done using SAS with the exception of logistic regression analyses, which were done using BMDP procedures.[19]

### RESULTS

Between November 1, 1984, and March 31, 1989, 13,914 women were enrolled into the VIP Study, of whom 13,750 (99%) had *C. trachomatis* results available. *C. trachomatis* was isolated from 1,239 (9.0%) women, 204 of whom were ineligible for the trial due to *N. gonorrhoeae* infection (n = 59), asymptomatic bacteriuria (n = 60), or other exclusion criteria. We were able to contact 933 of the 1,035 eligible women. Of those contacted, 218 women (23%) did not keep their enrollment appointment, 121 (13%) refused to participate, and 594 women were entered into the placebo run-in. The 180 women who did not comply with the run-in were not randomized, leaving 414 women who were randomized to receive either erythromycin (205) or placebo (209). After starting medication, 25 erythromycin-treated and 23 placebo-treated women withdrew from the trial but were included in the intent-to-treat analysis.

The two groups were compared with respect to demographic, behavioral, and obstetrical characteristics (Table 1). No significant differences were seen in baseline characteristics between the groups (Table 1). Additional factors that did not differ between groups included living arrangement; method/source of medical payment; work during pregnancy; number of sexual partners during pregnancy, in the last year, and lifetime; frequency of intercourse; prior genital or kidney infection; history of a cone biopsy; difficulty becoming pregnant; hospitalization during pregnancy; general health; illicit drug use; mean height and weight; past chlamydial or gonococcal infection or present genital infection (group B streptococci, *U. urealyticum*, *Trichomonas vaginalis*, bacterial vaginosis, or endocervical mucopus).

The pregnancy outcomes of women entered into the trial are summarized in Table 2. The mean birth weight of infants was similar in the two groups as was the distribution of birth weights. Birth weight <2,500 g occurred in 8% of erythromycin-treated and 11% of placebo-treated women ($P = 0.4$) Additionally, there were no differences in the distribution of gestational age, gestational age at the time of premature rupture of membranes (PROM), or the total proportion of women experiencing PROM. The numbers of stillbirths and neonatal deaths were low in both groups. Thus, when data from all study sites were combined, there was no statistically significant impact of erythromycin on pregnancy outcome, although there were fewer low birth weight infants, fewer deliveries <37 weeks gestation, and fewer instances of PROM in the erythromycin compared to the placebo group.

Compliance data were available for 199 of the 205 women in the erythromycin group and 206 of

**TABLE 1. Descriptive characteristics of patients with *C. trachomatis* randomized to receive either erythromycin or placebo**

| | Erythromycin (n = 205) | Placebo (n = 209) |
|---|---|---|
| Mean age ± S.D. (years) | 21.5 ± 4.2 | 21.1 ± 4.3 |
| Mean gravidity ± S.D. | 2.0 ± 1.2 | 1.9 ± 1.2 |
| Mean gestational age when screened ± S.D. (weeks) | 24.5 ± 1.1 | 24.5 ± 1.1 |
| Mean gestational age when randomized ± S.D. (weeks) | 29.4 ± 1.8 | 29.4 ± 1.5 |
| Ethnicity | | |
| White, Asian, and Native American | 34 (17%) | 33 (16%) |
| Black | 126 (61%) | 123 (59%) |
| New York Hispanic | 34 (17%) | 47 (22%) |
| Non-New York Hispanic | 11 (5%) | 6 (3%) |
| Marital status | | |
| Married | 59 (29%) | 48 (23%) |
| Separated/divorced/widowed | 18 (9%) | 21 (10%) |
| Never married | 127 (62%) | 140 (67%) |
| Education (years) | | |
| <12 | 90 (44%) | 97 (47%) |
| 12 | 84 (41%) | 73 (35%) |
| >12 | 30 (15%) | 38 (18%) |
| Smoked cigarettes during the pregnancy | 50 (24%) | 48 (23%) |
| Used alcohol during the pregnancy | 35 (17%) | 37 (18%) |
| Prior low birth weight delivery[a] | | |
| Yes | 46 (22%) | 52 (25%) |
| No | 70 (34%) | 55 (26%) |
| First pregnancy | 89 (43%) | 102 (49%) |
| Study site | | |
| Harlem Hospital | 6 (3%) | 9 (4%) |
| Columbia University | 54 (26%) | 59 (28%) |
| University of Washington | 6 (3%) | 7 (3%) |
| University of Oklahoma | 29 (14%) | 33 (16%) |
| University of Texas | 10 (5%) | 3 (1%) |
| Louisiana State University/ Tulane University | 100 (49%) | 98 (47%) |

[a]Included patients who delivered an infant weighing <2,500 with a prior pregnancy.

**TABLE 2. Effect of erythromycin treatment on pregnancy outcome in patients with *C. trachomatis* for the total group in an intent-to-treat analysis**

| | Outcome/total | |
|---|---|---|
| | Erythromycin (n = 205)[a] | Placebo (n = 209)[a] |
| Mean birth weight ± S.D. (g) | 3,192 ± 524 | 3,146 ± 552 |
| Low birth weight at delivery (g) | | |
| <1,500 | 0/201 | 2/199 (1%) |
| 1,500–2,499 | 17/201 (8%) | 20/199 (10%) |
| Total <2,500 | 17/201 (8%) | 22/199 (11%) |
| Gestational age at delivery (weeks) | | |
| <32 | 1/202 (0.5%) | 1/203 (0.5%) |
| 32–36 | 26/202 (13%) | 29/203 (14%) |
| Total <37 | 27/202 (13%) | 30/203 (15%) |
| Premature rupture of membranes (weeks) | | |
| <37 | 5/196 (3%) | 7/193 (4%) |
| ≥37 | 16/196 (8%) | 18/193 (9%) |
| Total PROM | 21/196 (11%) | 25/193 (13%) |
| Stillbirth | 2/202 (1%) | 1/203 (0.5%) |
| Neonatal death | 1/202 (0.5%) | 0/203 |

[a]The difference between the number of women enrolled in each arm of the treatment trial and the numbers assessed for each outcome reflect the numbers of women for whom data could not be obtained.

the 209 women in the placebo group. Twenty-three percent of the 199 erythromycin-treated women took less than two-thirds of their pills compared to 16% of the 206 placebo-treated women ($P = 0.053$). Nausea was the only side effect reported significantly more often among women in the erythromycin group (33%) compared to the placebo group (21%) ($P = 0.005$). Appetite loss was also more frequent in the erythromycin than the placebo group (21% vs. 14%, $P = 0.08$). Overall, 55% of women taking erythromycin experienced at least one side effect compared to 43% of women on placebo ($P = 0.01$). The effect of erythromycin on low birth weight and preterm delivery was stratified by compliance with no apparent effect on pregnancy outcome.

Endocervical *C. trachomatis* cultures obtained mid-study, while women were still receiving the study drug, remained positive in 20% of erythromycin-treated and 63% of placebo-treated women ($P < 0.001$). *C. trachomatis* was recovered from 8 (14%) of 56 erythromycin-treated women who took two-thirds or more of their pills compared to 7 (33%) of 19 women taking less than two-thirds of their erythromycin ($P = 0.03$).

Because of the unexpectedly high clearance of *C. trachomatis* in the placebo group (37%), the data were analyzed separately by study site (Table 3). The interim recovery rates were low (24–25%) in the placebo group at the New York and Oklahoma sites as opposed to the New Orleans and Seattle sites, where 89% and 83% of placebo-treated women continued to have positive cultures. Only one woman at San Antonio had repeat culture data.

In an attempt to explain these results, we first examined the repeatability of culture results from the quality control data. Recovery rates of *C. trachomatis* from quality control specimens were similar at the various sites. Overall, laboratories correctly identified 70 of 75 positive and 84 of 85 negative *C. trachomatis* quality control specimens.

**TABLE 3. Bacteriologic effect of erythromycin treatment measured by interim endocervical *C. trachomatis* cultures[a]**

| | No. *C. trachomatis* positive/No. treated | | |
|---|---|---|---|
| | Erythromycin | Placebo | *P* |
| New Orleans | 10/39 (25%) | 39/44 (89%) | <0.001 |
| Seattle | 0/4 | 5/6 (83%) | 0.048 |
| San Antonio | 0/1 | 0/0 | — |
| New York | 1/21 (5%) | 4/19 (24%) | 0.17 |
| Oklahoma | 5/13 (38%) | 3/12 (25%) | 0.67 |

[a]The two New Orleans sites and the two New York sites utilized the same laboratories and the data were combined.

The results of blinded split specimens were also in agreement. The proportion of samples for which both specimens were positive among the total with at least one positive specimen were: Louisiana State University/Tulane, 24/29 (83%); Columbia University/Harlem, 16/22 (73%); University of Oklahoma, 3/10 (30%); University of Washington, 0/1 (0%); and University of Texas 1/1 (100%).

We next examined data on the use of antibiotics prescribed outside of the clinical trial as a possible explanation for the high clearance rate in the placebo group. Significantly more use of non-trial antibiotics effective against *C. trachomatis* occurred in the placebo than the erythromycin group (22 vs. 8 women, $P < 0.01$). Harlem, Columbia, and Oklahoma showed a greater use of antibiotics in the placebo than the erythromycin group (33.3% vs. 0.0%, 17.0% vs. 1.9%, 18.8% vs. 3.5%, respectively). New Orleans had roughly similar rates in the two groups (5.6% vs. 2.8%), while San Antonio showed a greater use of non-trial antibiotics in the erythromycin group (0 vs. 20.0%). The number of women receiving effective antibiotics did not explain all of the differences in *C. trachomatis* positively among placebo-treated women.

The trial outcome data were then stratified into two groups: data from study sites with low vs. high *C. trachomatis* clearance in the placebo group. In the sites with low clearance (New Orleans, Seattle, and San Antonio), low birth weight infants occurred in 9 of 114 erythromycin-treated and 18 of 105 placebo-treated women ($P = 0.04$; Table 4) and delivery <37 weeks occurred in 15 of the 115 erythromycin and 18 of the 105 placebo group ($P = 0.4$). In the sites with high clearance (New York and Oklahoma), low birth weight occurred in 8 of 87 erythromycin-treated and 4 of 94 placebo-treated women ($P = 0.18$) and delivery <37 weeks occurred in 12 of 87 erythromycin-treated and 12 of 98 placebo-treated women ($P = 0.75$).

### DISCUSSION

In the intent-to-treat analyses, there were no apparent beneficial effects of erythromycin treatment on low birth weight, preterm delivery, PROM, or perinatal mortality. However, when the analysis took into account the clearance of *C. trachomatis* in the placebo group, erythromycin was associated with a 50% reduction in the rate of low birth weight in the centers with low clearance.

The higher rate of low birth weight in the placebo group in these sites is consistent with the increased rate of low birth weight observed among untreated women with *C. trachomatis* in other studies.[1,2,4,5,10] The proportion of infants with low birth weight in the erythromycin group (8%) was consistent with that for *C. trachomatis*-negative women in the observational component of the VIP Study (7.6%), and the proportion of low birth weight infants in the placebo group (11%) was similar to that among *C. trachomatis*-positive women in the VIP Study (10.6%) who were not entered into the trial.

There were at least three significant problems that interfered with our ability to detect an overall effect of erythromycin on pregnancy outcome. First, the trial included only 414 women, so the ability to detect small but potentially important differences between the erythromycin and placebo groups was limited. Second, approximately 20% of women receiving erythromycin remained *C. trachomatis* positive as determined from the random sample who were recultured. Third, at three study sites which contributed 46% of the cases to the trial, high clearance of *C. trachomatis* occurred in the placebo group.

The 20% failure rate of erythromycin in our study suggests the 1 g dose is less than optimal, possibly due to the 40% increase in blood and extracellular volume in pregnancy acting to reduce serum and tissue drug levels. While some investigators have reported cure rates in pregnancy of 98% with 1,500 mg of amoxicillin daily for 7 days,[20] and 95% with 1,000 mg of clindamycin daily for 14 days,[21] cure rates with erythromycin seem to be more variable, perhaps related to gastrointestinal tolerance and, in turn, to non-compliance.[20] *C. trachomatis* cure rates after erythromycin have ranged

TABLE 4. Stratification of trial outcome by treatment and study center

| Study site stratified by *C. trachomatis* clearance in the placebo group | Birth weight <2,500 g | | Delivery at <37 weeks | |
|---|---|---|---|---|
| | Erythromycin (n = 201) | Placebo (n = 199) | Erythromycin (n = 202) | Placebo (n = 203) |
| High clearance | | | | |
| New York-Harlem | 1/6 (17%) | 0/9 (0%) | 1/6 (17%) | 1/9 (11%) |
| New York-Columbia | 5/52 (10%) | 2/53 (4%) | 8/52 (15%) | 6/57 (11%) |
| Oklahoma City | 2/29 (7%) | 2/32 (6%) | 3/29 (10%) | 5/32 (16%) |
| Total | 8/87 (9%) | 4/94 (4%) | 12/87 (14%) | 12/98 (12%) |
| Low clearance | | | | |
| Seattle | 1/6 (17%) | 0/7 (0%) | 3/6 (50%) | 1/7 (14%) |
| San Antonio | 0/10 (0%) | 0/3 (0%) | 1/10 (10%) | 0/3 (0%) |
| New Orleans | 8/98 (8%) | 18/95 (19%) | 11/99 (11%) | 17/95 (18%) |
| Total | 9/114 (8%) | 18/105 (17%) | 15/115 (13%) | 18/105 (17%) |

from 92 to 95% with 1,600–2,000 mg daily doses for 7 days[20,22] to 76–88% with 1,000–2,000 mg daily doses for 7–14 days.[11,21] Direct comparisons of 1,000 vs. 2,000 mg daily doses of erythromycin have not been performed during pregnancy, but in non-pregnant women, one study has suggested that the 1,000 mg dose appears to be less effective than the 2,000 mg daily dose. *C. trachomatis* was recovered after therapy in 27% of 45 non-pregnant women given 1,000 mg and 10% of 31 non-pregnant women given 2,000 mg.[23] The low recovery rate in the placebo group was unexpected since other studies have shown that *C. trachomatis* persists in 85–90% of untreated non-pregnant[23,24] and 71–75% of untreated pregnant women.[20]

The effect of treating *C. trachomatis* has been reported in two studies. Ryan et al.[10] compared data on the pregnancy outcome of *C. trachomatis*-infected women treated with 2 g of erythromycin for 7 days with historical data on untreated women with *C. trachomatis* cared for in the previous 16 months in the same antenatal clinic. Treatment with erythromycin was associated with a 50% reduction of low birth weight, a 45% reduction of PROM, and a 400% increase in newborn survival.[10] Cohen et al.[11] reported the pregnancy outcome of women who were initially *C. trachomatis* positive and then underwent multiple repeat cultures after standard erythromycin therapy to detect treatment failures and reinfection. Low birth weight, PROM, and preterm delivery were more common in the women who remained positive than in the women who became *C. trachomatis* negative.[11] Neither of these studies employed a randomized trial design.

What conclusions may be drawn from the studies of *C. trachomatis* and pregnancy outcome to date? Data from our observational study suggest an impact of untreated *C. trachomatis* on low birth weight, preterm delivery, and preterm PROM. Results of site-specific analyses from our clinical trial together with the results of Ryan et al.[10] and Cohen et al.[11] suggest that the risk of low birth weight in *C. trachomatis*-positive women can be decreased by erythromycin treatment.

Even in the absence of incontrovertible evidence of a role of *C. trachomatis* in abnormal pregnancy outcome, there is a strong rationale for the antepartum diagnosis and treatment of *Chlamydia.* First, treatment prevents transmission of *C. trachomatis* to the infant at delivery. The 15–20% rate of chlamydial conjunctivitis and chlamydial pneumonia[25] appears to be reduced by 90% when the mother is treated.[20,22] Second, treatment reduces the spread to sexual partners. Third, treatment can prevent fallopian tube damage and possible future tubal infertility among women who otherwise would be chronically infected or develop salpingitis postpartum.[26]

Therefore, antepartum screening and treatment of *C. trachomatis* are recommended by the Centers for Disease Control (CDC).[27] Although treatment at 36 weeks of pregnancy would prevent vertical transmission to the 90% of infants born beyond this time,[20,22] screening and treatment late in pregnancy would not be expected to prevent premature delivery. Treatment in the mid-trimester could both potentially reduce premature delivery rates and protect the infant from vertical transmission. In our opinion, these considerations tip the balance in favor of mid-trimester as the optimal time to treat *C. trachomatis* as opposed to the later time recommended by the CDC.[27]

### ACKNOWLEDGMENTS

This work was supported by contracts (HD-3-2832 through HD-3-2836) from the National Institute of Child Health and Human Development and the National Institute of Allergy and Infectious Diseases (AI-4-2532).

### REFERENCES

1. Martin DH, Koutsky LA, Eschenbach DA, Daling JR, Alexander ER, Holmes KK: Perinatal mortality and prematurity in pregnancies complicated by antepartum maternal *Chlamydia trachomatis* infection. JAMA 247:1585–1588, 1982.
2. Sweet RL, Landers DV, Walker C, Schachter J: *Chlamydia trachomatis* infection and pregnancy outcome. Am J Obstet Gynecol 156:824–833, 1987.
3. Heggie AD, Lumicao GG, Stuart LA, Gyves MT: *Chlamydia trachomatis* infection in mothers and infants. Am J Dis Child 135:507, 1981.
4. Gravett MG, Nelson HP, DeRouen T, Critchlow CW, Eschenbach DA, Holmes KK: Independent association of bacterial vaginosis and *Chlamydia trachomatis* infection with adverse pregnancy outcome. JAMA 256:1899–1903, 1986.
5. Alger LS, Lovchik JC, Hebel JR, Blackmon LR, Crenshaw MC: The association of *Chlamydia trachomatis, Neisseria gonorrhoeae,* and group B streptococci with preterm rupture of the membranes and pregnancy outcome. Am J Obstet Gynecol 159:397–404, 1988.
6. Shafer MA, Beck A, Blain B, et al.: *Chlamydia trachomatis:* Important relationships to race, contraceptive use, lower genital tract infection and Papanicolaou smears. J Pediatr 104:141–146, 1984.
7. Eager RM, Beach RK, Davidson AJ, Judson FN: Epidemiologic and clinical factors of *Chlamydia trachomatis* in black, Hispanic and white female adolescents. West J Med 143:37, 1985.
8. Harrison HR, Alexander ER, Weinstein L, Lewis M, Nash M, Sim DA: Cervical *Chlamydia trachomatis* and mycoplasmal infections in pregnancy: Epidemiology and outcomes. JAMA 250:1721–1727, 1983.
9. Berman SM, Harrison HR, Boyce WT, Haffner WJ, Lewis M, Arthur JB: Low birth weight, prematurity, and postpartum endometritis. Association with prenatal cervical *Mycoplasma hominis* and *Chlamydia trachomatis* infections. JAMA 257:1189–1194, 1987.
10. Ryan GM, Abdell RN, McNeeley G, et al.: *Chlamydia trachomatis* infection in pregnancy and effect of treatment on outcome. Am J Obstet Gynecol 162:34–39, 1990.
11. Cohen I, Veille J, Colkins BM: Improved pregnancy outcome following successful treatment of chlamydial infection. JAMA 263:3160–3163, 1990.
12. Klebanoff MA, Regan JA, Rao AV, et al.: Outcome of the Vaginal Infections and Prematurity Study: Results of a clinical trial of erythromycin among pregnant women colonized with group B streptococci. Am J Obstet Gynecol 172:1540–1545, 1995.
13. Eschenbach DA, Nugent RP, Rao AV, et al., and Vaginal Infections and Prematurity Study Group: A randomized placebo-controlled trial of erythromycin for the treatment of *U. urealyticum* to prevent premature delivery. Am J Obstet Gynecol 164:734–742, 1991.
14. Schachter J: *Chlamydiae.* In Balows A, Hausler WJ, Herrmann KL, Isenberg HD, Shadomy HJ (eds): Clinical Microbiology. 5th ed. Washington, DC: American Society for Microbiology, 1991.
15. Stamm WE, Tam MR, Koester M, Cles L: Detection of *Chlamydia trachomatis* inclusions in McCoy cell cultures with fluorescein-conjugated monoclonal antibodies. J Clin Microbiol 17:666–668, 1983.
16. Blackwelder WC, Hastings BK, Lee MF, et al.: Value of a run-in period in a drug trial during pregnancy. Controlled Clin Trial 11:187–198, 1990.
17. McCormack WM, Rosner B, Lee Y, Munoz A, Charles D, Kass EH: Effect on birth weight of erythromycin treatment of pregnant women. Obstet Gynecol 69:202–208, 1987.
18. Armitage P, Berry G (eds): Statistical Methods in Medical Research. Boston: Blackwell Scientific, pp 372–374, 1987.
19. SAS Institute: SAS User's Guide. Version 5. Cary, NC: SAS Institute, 1985.
20. Crombleholme W, Schachter J, Grossman M, Landers DV, Sweet RL: Amoxicillin therapy for *Chlamydia trachomatis* in pregnancy. Obstet Gynecol 75:752, 1990.
21. Alger LS, Lovchik JC: Comparative efficacy of clindamycin versus erythromycin in eradication of antenatal *Chlamydia trachomatis.* Am J Obstet Gynecol 165:375–381, 1991.
22. Schachter J, Sweet RL, Grossman M, et al.: Experience with the routine use of erythromycin for chlamydial infections in pregnancy. N Engl J Med 314:276–279, 1986.
23. Linnemann CC, Heaton CL, Ritchey M: Treatment of *Chlamydia trachomatis* infections: Comparison of 1- and 2-g doses of erythromycin daily for seven days. Sex Transm Dis 14:102–106, 1987.
24. Handsfield HH, Murphy VL: Treatment of uncomplicated gonorrhea in women with single-dose cefonicid. Sex Transm Dis 12:90–92, 1985.
25. Schachter J, Grossman M, Sweet RL, et al.: Prospective study of perinatal transmission of *Chlamydia trachomatis.* JAMA 255:3374–3377, 1986.
26. Plummer FA, Laga M, Brunham RC, et al.: Postpartum upper genital infections in Nairobi, Kenya: Epidemiology, etiology and risk factors. J Infect Dis 157:92–98, 1987.
27. U.S. Public Health Service Centers for Disease Control: Sexually transmitted disease treatment guidelines. MMWR 38(S-8):1–40, 1989.
